# A model for the geomagnetic field reversal rate and constraints on the heat flux variations at the core-mantle boundary

**DOI:** 10.1038/s41598-020-69916-w

**Published:** 2020-08-03

**Authors:** Vincenzo Carbone, Tommaso Alberti, Fabio Lepreti, Antonio Vecchio

**Affiliations:** 1 Dipartimento di Fisica, Università Della Calabria, Ponte P. Bucci, Cubo 31C, 87036 Rende, CS Italy; 2 Istituto Nazionale di Astrofisica (INAF), Direzione Scientifica, Rome, Italy; 3 INAF-IAPS Istituto di Astrofisica e Planetologia Spaziali, Via Fosso del Cavaliere 100, 00133 Rome, Italy; 4 Radboud Radio Lab, Department of Astrophysics/IMAPP, Radboud University, P.O. Box 9010, 6500GL Nijmegen, The Netherlands; 5 LESIA - Observatoire de Paris, Université PSL, CNRS, Sorbonne Université, Université de Paris, 5 place Jules Janssen, 92195 Meudon, France

**Keywords:** Planetary science, Core processes

## Abstract

A striking feature of many natural magnetic fields generated by dynamo action is the occurrence of polarity reversals. Paleomagnetic measurements revealed that the Earth’s magnetic field has been characterised by few hundred stochastic polarity switches during its history. The rate of reversals changes in time, maybe obeying some underlying regular pattern. While chaotic dynamical systems can describe the short-term behaviour of the switches of the Earth’s magnetic polarity, modelling the long-term variations of the reversal rate is somewhat problematic, as they occur on timescales of tens to hundreds of millions of years, of the order of mantle convection timescales. By investigating data of geomagnetic reversal rates, we find the presence of cycles with variable frequency and show that the transition towards periods where reversals do not occur for tens of million years (superchrons) can be described by a second-order phase transition that we interpret to be driven by variations of the heat flux at the core-mantle boundary (CMB). The model allows us to extract from the reversal sequence quantitative information on the susceptibility of the reversal rate caused by changes in the CMB heat flux amplitude, thus providing direct information on the deep inner layers of the Earth.

## Introduction

The Earth’s magnetic field is generated, through the dynamo effect, by the motion of liquid metal in the core^1^. Paleomagnetic measurements revealed that the dipole component of the magnetic field has undergone several polarity switches during the Earth’s history, the periods spent in a single polarity being stochastically distributed^2-4^. The occurrence of polarity reversals is a common property of the dynamo phenomenon being observed also in the Sun^5^, even though there the magnetic field reverses quasi periodically every ~ 11 years^6,7^, and in laboratory excited dynamo^8^. The physical mechanism which drives the Earth’s magnetic field to reverse is still controversial. Some simple dynamical systems can describe the reversal dynamics^9,10^ up to timescales of several Myr (longer than the core magnetic diffusion time of about 10^5^ yr), and reversals have been observed in 3D global direct numerical simulations^11-14^. Although statistical inferences of Earth’s magnetic field reversals are conditioned by their relatively small number, it has been argued that they can be described as a sequence of stochastic independent events^15^, thus following a Poisson statistics^16,17^. As a different point of view, it has been shown that the sequence of periods spent in a single magnetic polarity is characterised by the presence of long-range correlations^18,19^ and that it can be better described by a Lévy process^20^. The statistical difference could be ascribed to the fact that, on long time scales, the rate of reversals is not constant^17^, showing transitions from periods of rapid polarity switches to very long intervals of fixed polarity, called superchrons and lasting for a few tens of Myr, such as occurred between the Middle Jurassic and Middle Cretaceous periods. Data analysis of polarity reversals revealed that different cycles seem to be present in the variations of the rate, whose periods are related to typical time scales of mantle convection processes^21-27^. On this basis, it has been suggested that the changes of the reversal rate may have been triggered by fluctuations in CMB heat flow, either global or localised in equatorial regions^23,28-30^. In several works based on geodynamo numerical simulations, the effects of CMB heat flux amplitude and morphology have been investigated and discussed in the context of observed reversal rate changes^31-36^.

## Results

**Figure 1:**
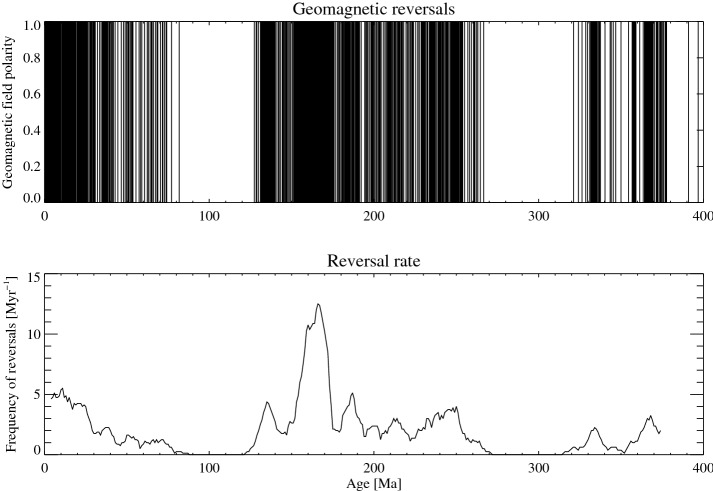
Upper panel: geomagnetic polarity back to 375.3 Ma. The polarity is denoted by 1 for the present polarity and 0 for reversed. A line connects the values, therefore black regions correspond to a high reversal rate and white regions to intervals of constant value. Lower panel: reversal rate time series obtained by averaging over sliding windows of length *Δt* = 8 Myr (see Supplementary Information).

**Figure 2:**
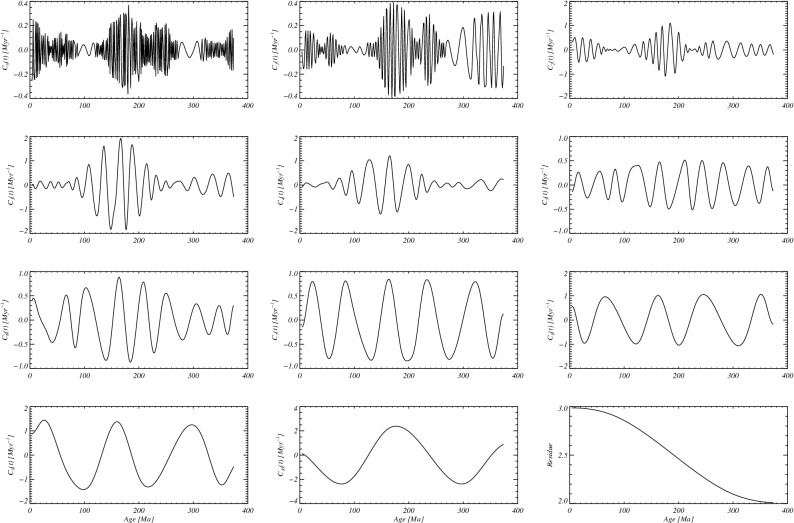
IMFs obtained through the EMD technique applied to the reversal rate time series. The residue of the decomposition is shown in the lowest right panel, while the IMFs *C*_*j*_(*t*), for *j* = 0,1, …, 10, are reported in the other panels.

We analyse data^26^ obtained from the latest geomagnetic polarity time scale (2012), integrated with data from the late Devonian^37^. The time sequence of polarity reversals was reconstructed for the whole Phanerozoic eon^26^. The time sequence of reversals and the time evolution of the rate of reversals *γ*(*t*), obtained by averaging reversals over sliding windows of width 8 Myr, are reported in Fig. 1. We analysed the geomagnetic series *γ*(*t*) in terms of empirical modes, through the Empirical Mode Decomposition (EMD, see “Methods”), a technique which is particularly suitable to process non-stationary time series^38^ like the reversal rate series. Through this technique, a time series is decomposed into empirical modes, called Intrinsic Mode Functions (IMFs), characterized by different frequencies and therefore it is possible to extract relevant timescales involved in the non-stationary process under investigation. For the reversal rate time series *γ*(*t*) we obtained a sequence of 11 IMFs *C*_*j*_(*t*) (*j* = 0,1, …, 10), which are shown in Fig. 2 along with the residue (see “Methods”). For each mode we can extract an instantaneous frequency *ω*_*j*_(*t*), whose time variations describe the non-stationary processes underlying the observed variability of the reversal rate, and the time average of *ω*_*j*_(*t*) allows us to define a typical period *T*_*j*_ = 2π/〈*ω*_*j*_〉 (where the time average is denoted by angular brackets) associated to each *j*-th mode.

The probability density functions of the instantaneous periods 2π/*ω*_*j*_(*t*) (see Fig. S1 of Supplementary information) indicate a compatibility with previously reported cycles. Periods shorter than about 40 Myr^21,22,24,26,39^ are found in the modes with *j ≤ *5. Such variability has been related to CMB heat flux changes and plume dynamics within the Earth’s mantle. Modes characterized by longer periodicities, associated by our analysis to EMD modes with lower frequencies, are perhaps hidden in the low-frequency Fourier peaks commonly used to recover reversal rate periodicities. Previously reported periodicities also include time scales longer than 100 Myr, which can arise from subducting lithospheric slabs reaching the CMB^27,30^. These time scales are compatible with the instantaneous periods found in the modes *j* = 9,10. Moreover, note that the residue of the EMD (see Fig. 2) is a decreasing function of age, thus indicating that the global rate of reversals is not constant, and the global average persistence of the geomagnetic field in a single polarity state decreased, on average, going from 400 Ma to the present.

EMD results suggest that the geomagnetic system, for timescales longer than the magnetic diffusion time, can be modelled through transitions between chrons induced by a continuous underlying stochastic process, different chrons being characterized by the average frequency of switches *T*_*j*_^−1^ between different polarity states of the magnetic field. To identify the number of states present in the system, we describe the reversal rate variations in terms of a stochastic dynamical system and we assume that a transition among states of different reversal rates is triggered by a stochastic forcing, namely the continuous change of heat flux at CMB. We use the Langevin equation *dx* = -*U*′(*x*)*dt* + *σ dw* to describe the dynamics of the rate changes, where *x* is a state variable, which in our case represents an IMF or a sum of IMFs, *U*(*x*) is a given potential, *U*′(*x*) = *dU*/*dx*, *σ* is the noise level and *dw* is a Wiener process, i.e., a stochastic process with independent Gaussian increments, which describes the stochastic CMB heat flux. The potential function *U*(*x*) can be evaluated by means of the Fokker–Planck (FP) equation associated with the Langevin model, describing the time evolution of the probability density function (pdf) *p*(*x*,*t*) (see “Methods”). Moreover, if the potential function *U*(*x*) can be written as a polynomial function of even order *k* and positive leading coefficient, i.e., *U*(*x*) = *u*_*o*_ + *u*_1_ *x* + *u*_2_ *x*^2^ + *…* + *u*_*k*_* x*^*k*^ and *u*_*k*_ > 0, its order is related to the number of available states for the reversal rates *n*, i.e., *n* = *k*/2 ^40,41^. In this way, from the pdfs of the IMFs *C*_*j*_ we can calculate the potentials for all the EMD modes (see Fig. S3 of Supplementary information) by assuming that the stochastic term, i.e., the Wiener process, is representative of processes occurring at timescales which are shorter than the mean timescale *T*_*j*_ of each empirical mode *C*_*j*_, and that the amplitude of the noise corresponds to the standard deviation of each *C*_*j*_^40,41^. Two types of potential shapes are present in the dataset, thus reflecting the number of possible states for the reversal rate at different time scales *T*_*j*_. Namely, single-well potentials related to high-frequency CMB changes, for the set of modes *H* = {0 ≤ *j* ≤ 4}, and double-well potentials, related to low-frequency CMB changes, for the set of modes *L* = {5 ≤ *j* ≤ 10}. Some of the EMD modes corresponding to single-well potentials, namely the modes *j* = 2,3,4, present periodicities (between 14 and 30 Myr, see Table T1 of Supplementary Information) that are close to cycles already identified using different techniques^21,22,24,26,39^, as already mentioned above. The period of the EMD mode *j* = 10 with a double-well potential is close to what has already been observed as a superchron period^25^. In addition, the EMD analysis suggests the presence of characteristic intermediate time scales, in the range 50–100 Myr (see Table T1 of Supplementary Information). These periods, corresponding to asymmetric double-well potentials, are perhaps hidden in the large width of low-frequency Fourier modes reported in previous analyses^25,26^.

The approach based on stochastic Langevin models has been proven successful in reproducing the dipole field variability observed both in paleomagnetic data covering the last 2 Myr^42,43^ and in numerical geodynamo simulations^43^. It has been shown that such models provide a good description of the axial dipole field dynamics and a reliable prediction, through the stationary solution of the FP equation, of its probability distribution. Here, we follow a similar approach and we assess the significance of our Langevin model by comparing, firstly, the partial reconstruction of the geomagnetic reversal rate signal obtained by summing the IMFs of the set of modes *L* = {5 ≤ *j* ≤ 10} to a realisation obtained from the stochastic Langevin model (see Fig. S5 of Supplementary Information), and, then, the stationary solution of the FP equation to the pdfs of the partial reconstruction and of the Langevin model (see Fig. S6 of Supplementary Information). A quite good agreement is found between the pdfs, thus confirming the validity of our approach.

**Figure 3:**
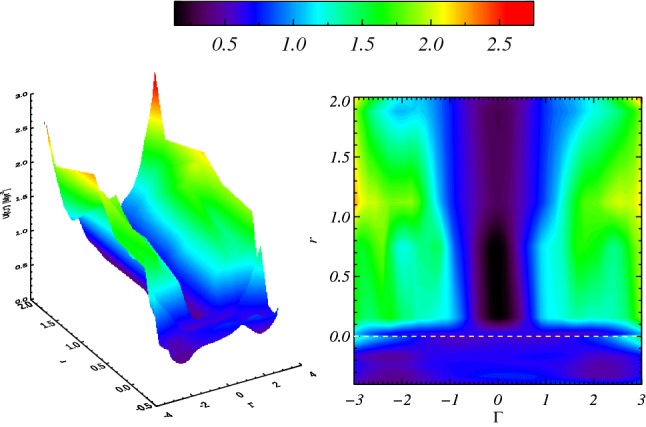
The left panel shows the potential manifold *U*(*r*,*Γ*), the right panel shows the manifold projected on the (*r*,*Γ*) plane. The manifold has been drawn by connecting the potentials obtained for each EMD mode. The parameters *u* and *h* are obtained through best fits on the potentials obtained from the empirical modes.

**Figure 4:**
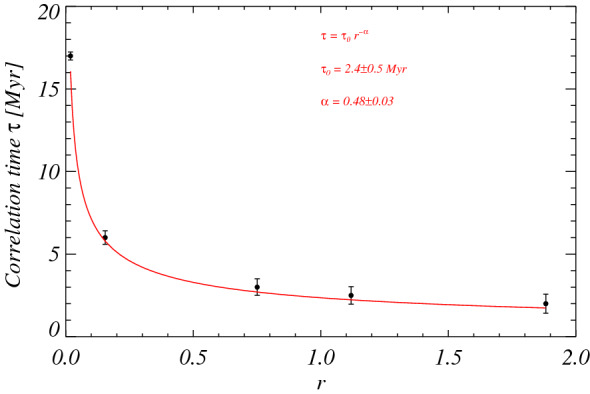
Correlation time *τ* (black circles), estimated from the two-times correlation coefficient of the observed reversal rates for empirical modes from *j* = 6 to *j* = 10, as a function of the scaling parameter *r*. The superposed red curve corresponds to a fit obtained through an inverse power law function, reported on the figure along with the best fit parameters.

The dynamics of the obtained EMD modes allows us to interpret the transition from high-frequency chrons towards low-frequency superchrons as a kind of phase transition^44^, that we assume to be driven by stochastic fluctuations of the heat flux at the CMB. High-frequency chrons correspond to disordered states characterized by periods of rapid polarity reversals and stronger CMB activity. On the contrary, low-frequency rates correspond to more organized states, characterized by stable long residence times in a single magnetic polarity, with smaller CMB heat flux variations and weaker mantle plume activity. In the framework of mean-field approximation^44^, let us consider a continuous order parameter from the set of standardised EMD modes $\Gamma = \left\{ {C_{j}^{\sigma } \left( t \right)} \right\}$ (see Supplementary Information) and let us write up the potentials in terms of the manifold

1 $$ U\left( \Gamma \right) = r\Gamma^{2} + u\Gamma^{4} - h\Gamma $$

shown in Fig. 3. The transition from the single-well potential to the asymmetric double-well one, happens when the parameter *r* changes sign. Then we define *r* in terms of CMB heat flow as *r* = (*Q—Q*_*c*_)/*Q*_*c*_, where *Q*_*c*_ represents a critical threshold. In other words, according to the mean field approach of phase-transitions^44^, we assume that the transition to superchrons happens when the CMB heat flow *Q* becomes smaller than the critical value *Q*_*c*_. The correlation time *τ*, estimated from the two-times correlation coefficient *G*(*t*_1_, *t*_2_) of the observed reversal rates for each mode

2 $$ G\left( {t_{1} ,t_{2} } \right) = \Gamma \left( {t_{1} } \right)\Gamma \left( {t_{2} } \right) \approx \exp \left[ {\frac{{ - \left| {t_{1} - t_{2} } \right|}}{\tau }} \right], $$

(where the angular brackets denote time averaging) shows a power law dependence *τ* ≈ 1/*r*^*α*^ and thus diverges when *Q* ≈ *Q*_*c*_ (see Fig. 4), where *α* = 0.48 ± 0.03, in close agreement with the scaling exponent *α* = 1/2 required by the mean-field approximation of second-order phase transitions^44^. This confirms that a kind of second-order phase transition is at work within the complex geodynamo system.

**Figure 5:**
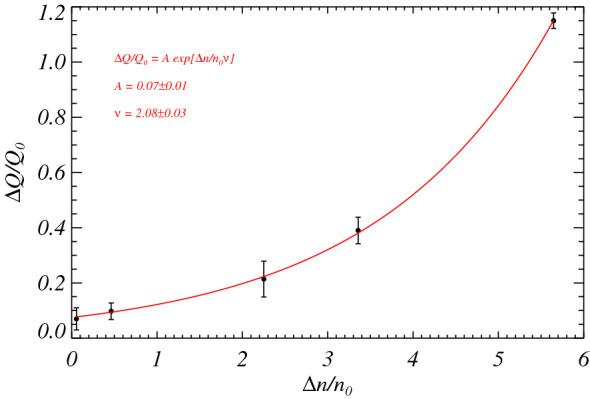
Estimated excess of CMB heat flow (black circles) as a function of the expected relative increase of reversals. The superposed red curve corresponds to an exponential function, obtained through a fit on the data, reported on the figure along with the best fit parameters.

According to the mean field approximation of second-order phase transitions, the susceptibility *χ* is given by *χ*^−1^ = *dh*/*dΓ*. The equilibrium solution in the mean field approach is obtained by minimising the potential *U* (Eq. (1)) with respect to *Γ.* This gives *h* = 2*rΓ* + 4*uΓ*^3^, from which *χ*^−1^ = 2*r* + 12*uΓ*^2^. In the mean field theory, for the single-well minimum, which corresponds to the disordered phase when the geomagnetic field reverses at a high rate, we have *Γ*^2^ = 0 when *r* > 0, therefore *χ*^−1^ = 2*r.* This result is very interesting in our case, as it allows us to infer the number of reversals induced, starting from a reference value *n*_0_, by amplitude variations of the heat flux at CMB. In fact, since the heat flux variations due to a variation of the order parameter must be roughly proportional to the variations of the *h* field of the model, namely *ΔQ ≈ Δh*, we get *ΔQ ≈ χ*^−1^ *ΔΓ ≈* 2*r ΔΓ*. By estimating *ΔΓ* as *ΔΓ ≈* 1/(*T*_*j*_*—T*_*j-*1_), using the characteristic periods *T*_*j*_ of EMD modes *j* = 6–10, we can use the predictive property of the susceptibility to directly infer about the heat flow fluctuations at CMB, relative to a reference value *Q*_0_, required to increase the reversal rate from the reference value *n*_0_, as shown in Fig. 5. An empirical relation can be obtained through a fit on the data with the following exponential function

3 $$ \frac{\Delta Q}{{Q_{0} }} = A\exp \left[ {\frac{\Delta n}{{n_{0} \nu }}} \right] $$

where *Δn* is the variation in the reversal rate and the best fit parameters result *ν* = 2.08 ± 0.03 and *A* = 0.07 ± 0.01. This means that fluctuations of the order of about *ΔQ ≈* 0.18 *Q*_0_ of CMB heat flow should be enough to double the reversal rate in the geodynamo system.

## Discussion

**Figure 6:**
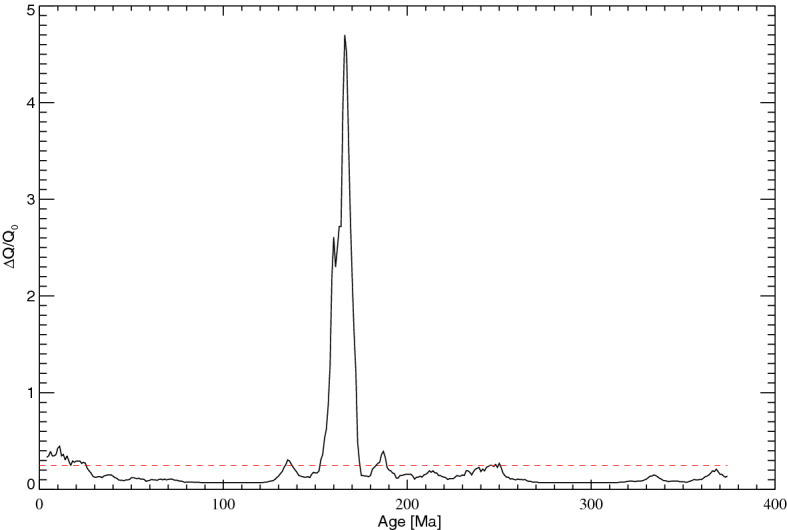
Estimated relative CMB heat flux variations over the last 375 Myr obtained from the observed reversal rate variations by using Eq. (3). The dashed red line corresponds to the average CMB flux.

It is worth remarking that our model represents a novel conceptual way of approaching geodynamo reversals over the last 400 Myr, by using the existing reversal dataset. This is very interesting because it allows us to provide constraints, directly from the observed geomagnetic reversal sequence, on the expected CMB heat flux variations. Using Eq. (3) with *n*_0_ = 1.43 Myr^−1^, which is the median value of the reversal frequency over the interval covered by the considered dataset, and assuming *Δn* = *γ*(*t*), in Fig. 6 we report the expected evolution of the heat flux variations required to account for the observed reversal rate. The spike between 160 and 170 Ma corresponds to the peak of the reversal rate in the same interval. It is worthwhile to point out that the number of reversals occurring in this interval is likely to be overestimated (by a factor up to about 2) because some anomalies caused by geomagnetic field fluctuations could be incorrectly interpreted as reversals^45,46^. This could significantly reduce the amplitude of the spike in the reversal rate and, consequently, also the corresponding spike in *ΔQ*/*Q*_0_, however without making them to be suppressed.

By using Eq. (3) to make a comparison with the heat flux variation at CMB of numerical simulations, we can estimate *Δn*/*n*_0_ ≈ 7–8 when *ΔQ*/*Q*_0_ ≈ 2–3. This is in agreement with direct estimates of the CMB heat flux evolution obtained using numerical geodynamo models with core evolution^30,47^, which show that an increase of about 10 reversals, starting from few reversals, is achieved if the CMB heat flux varies by a factor of 2 or 3. Also simpler purely chemically-driven dynamos without core evolution seem to be compatible with the results presented here. For example, the simulations of Driscoll and Olson^35^ show that variations in the thermo-chemical CMB buoyancy flux of a factor 2 can lead to a change from minor reversal activity (with reversal frequency less than ~ 1 Myr^−1^) to frequently reversing activity with frequency ~ 4.5 Myr^−1^. Olson et al.^34^ considered a tomographic forcing of the CMB heat flux, finding that variations in its mean amplitude of a factor 2 lead to a relative increase of reversal frequency of a factor 6–10. It has also been found that the rate of reversals scales with the buoyancy-flux-based Rayleigh number *Ra*^48^. Since the Rayleigh number is directly related to the heat flux at CMB, our relation (3) could be used to estimate values of *Ra* on varying the fluctuations of the number of reversals and provide constraints on this dimensionless parameter. It is also worth to mention here the following still open research problem in this context. Mantle convection models predict temporal variations in total CMB heat flux of the order of few tens of percent over the last 400 Myr^30,49^. According to the dynamo simulation results discussed above and the stochastic model presented here, such variations are unlikely to produce significant changes in reversal frequency.

As a conclusion, we introduced a conceptual model for the reversal rate of the geomagnetic field, based on a second-order phase transition driven by stochastic fluctuations, which allows us to recover the CMB heat flux time variations which drove the reversals over the past 400 Myr. The CMB heat flux fluctuations are derived from the available reversal dataset and can be used to infer a constraint on the variations required to increase the number of geomagnetic reversals by a given amount.

## Methods

### Empirical mode decomposition

The time evolution of the reversal rate *γ*(*t*) has been analysed through the Empirical Mode Decomposition (EMD)^38^. This decomposition is orthogonal and complete, and partial summations of EMD modes capture typical processes happening over different time scale ranges. In addition, EMD is able to avoid some limitations present in other decomposition analysis techniques. Differently from Fourier or wavelet analysis, EMD does not require any a priori assumption on the functional form of the basis of the decomposition. This allows us to extract local nonstationarity and nonlinearity features from each time series.

Through this technique the reversal rate time series has been decomposed as

4 $$ \gamma \left( t \right) = \mathop \sum \limits_{j = 0}^{m} C_{j} \left( t \right) + r_{m} \left( t \right) $$

where each Intrinsic Mode Function (IMF) *C*_*j*_(*t*) represents an oscillating function with both amplitude and phase modulation in time *C*_*j*_(*t*) = *A*_*j*_(*t*) cos[*ϕ*_*j*_(*t*)], while *r*_*m*_(*t*) is the residual of the decomposition, obtained after all the oscillating functions have been extracted. The various IMFs are extracted recursively using a sifting procedure^38^. As a first step the sets of local maxima and minima of *γ*(*t*) are identified and interpolated by cubic splines, thus obtaining an upper and a lower envelope, respectively, as well as their local mean *m*_1_(*t*). The difference *H*_1_(*t*) = *γ*(*t*) − *m*_1_(*t*) represents the first IMF if it satisfies two conditions: (1) the number of extrema and zero-crossings does not differ by more than 1; (2) the mean value of the envelopes obtained from the local maxima and minima is zero at any point. If these conditions are not fulfilled, the procedure is repeated on the *H*_1_(*t*) time series, and a new difference *H*_11_(*t*) = *H*_1_(*t*) − *m*_11_(*t*) is obtained, where *m*_11_(*t*) is the mean of the new envelopes. The process is iterated until the *s*-th iteration *H*_1*s*_ fulfils the IMF properties. To avoid loss of information about amplitude and frequency modulations, a stopping criterion between two consecutive iterations is introduced through the parameter

5 $$ \eta = \mathop \sum \limits_{t = 0}^{{T_{\max } }} \left[ {\frac{{\left| {H_{{1\left( {s - 1} \right)}} \left( t \right) - H_{1s} \left( t \right)} \right|^{2} }}{{H_{{1\left( {s - 1} \right)}}^{2} \left( t \right)}}} \right] $$

where *T*_*max*_ denotes the last time instant of the dataset. When *η* < *η*_*cr*_, with the threshold fixed at *η*_*cr*_ = 0.3 in our case, the process is stopped. When the first IMF is calculated, the first residue *r*_1_(*t*) = *γ*(*t*) − *C*_1_(*t*) is processed again, thus obtaining *C*_2_(*t*) and *r*_2_(*t*). The whole procedure is carried on until *C*_*m*_(*t*) or *r*_*m*_(*t*) are almost constant, or *r*_*m*_(*t*) is monotonic. From our time series we extracted 11 IMFs (*m* = 10) along with the residual *r*_*m*_(*t*). It is worthwhile to remark that for each IMF we can extract the instantaneous frequencies *ω*_*j*_(*t*) = *dϕ*_*j*_/*dt*, from which we can obtain the instantaneous periods 2π/*ω*_*j*_(*t*). The time averages of the frequencies *ω*_*j*_(*t*) allows us to define the typical periods *T*_*j*_ = 2π/〈*ω*_*j*_〉 associated to each mode.

### Langevin stochastic model

The statistical properties of IMFs can be described by using a simple stochastic model based on a Langevin process

6 $$ dx = - U^{{\prime }} \left( x \right)dt + \sigma dw $$

where *x* is the state variable (in our case an IMF or a sum of IMFs), *U* is the potential function, acting as a drift term, *σ* is the noise level, and *dw* is a stochastic process. Specifically, the stochastic process, mimicking the dynamics on timescales shorter than the characteristic period of each IMF, is a Wiener process, defined as a stochastic process with independent Gaussian increments, i.e., *dw* is normally distributed with zero mean and variance *t* (also called Brownian motion due to its historical connection with the related physical process). A very similar approach has been employed to describe the temporal behaviour of the dipole field both in paleomagnetic data and numerical geodynamo models^42,43^. The potential function *U*(*x*) can be derived from the stationary solution of the associated Fokker–Planck (FP) equation to the Langevin model which reads

7 $$ \frac{{\partial p\left( {x,t} \right)}}{\partial t} = \frac{ - \partial }{{\partial x}}\left[ {U^{\prime}\left( x \right)p\left( {x,t} \right)} \right] + \frac{{\sigma^{2} }}{2}\frac{{\partial^{2} }}{{\partial x^{2} }}\left[ {p\left( {x,t} \right)} \right] $$

where *p*(*x*,*t*) is the probability density function (pdf). By searching for a stationary solution of the FP equation, we obtain *-∂*/*∂x* {[*U*′(*x*) *p*(*x*,*t*)] + *σ*^2^/2 *∂*/*∂x*[*p*(*x*,*t*)]} = 0, whose solution is

8 $$ p\left( x \right) = p_{0} \exp \left[ {\frac{ - 2U\left( x \right)}{{\sigma^{2} }}} \right], $$

where *p*_0_ is a normalization factor. Thus, by deriving the pdf of each IMF we can get the corresponding potential function as

9 $$ U_{j} \left( x \right) = \frac{{ - \sigma^{2} }}{2}\log \left[ {\frac{{p_{j} \left( x \right)}}{{p_{0} }}} \right] $$

being *p*_*j*_(*x*) the empirical pdf of each IMF and choosing *p*_0_ such that *p*_*j*_(*x*) has a unit integral. Moreover, if the potential function *U*(*x*) can be written as a polynomial function of even order *k* and positive leading coefficient, i.e., *U*(*x*) = *u*_*o*_ + *u*_1_ *x* + *u*_2_ *x*^2^ + *…* + *u*_*k*_* x*^*k*^ and *u*_*k*_ > 0, its order is related to the number of available states for the reversal rates *n*, i.e., *n* = *k*/2^40,41^.

## Supplementary information

Supplementary Information.

## Data Availability

The data analysed in this work are available in the Supplementary materials of Melott, A.L., Pivarunas, A., Meert, J.G. & Lieberman, B.S. Does the planetary dynamo go cycling on? Re-examining the evidence for cycles in magnetic reversal rate. *International Journal of Astrobiology*, 10.1017/S1473550417000040 (2017).
