# Supplementary material for: A model for the geomagnetic field reversal rate and constraints on the heat flux variations at the core-mantle boundary
Source: Sci Rep. 2020 Aug 3;10:13008. doi: 10.1038/s41598-020-69916-w (PMC7398921; doi:10.1038/s41598-020-69916-w)
Supplement: Supplementary file 1 — Supplementary Information. [file 41598_2020_69916_MOESM1_ESM.pdf]

A model for the geomagnetic field reversal rate and constraints on the heat flux variations at the core-mantle boundary

Supplementary information

Vincenzo Carbone[1,2*], Tommaso Alberti[3], Fabio Lepreti[1,2], Antonio Vecchio[4,5]
* *Email-address*: vincenzo.carbone@fis.unical.it

[1]Dipartimento di Fisica, Università della Calabria, Ponte P. Bucci, Cubo 31C, 87036 Rende (CS), Italy
[2]Istituto Nazionale di Astrofisica (INAF), Direzione Scientifica, Roma, Italy
[3]INAF-IAPS Istituto di Astrofisica e Planetologia Spaziali, Via Fosso del Cavaliere 100, 00133 Roma (Italy)
[4]Radboud Radio Lab, Department of Astrophysics/IMAPP-Radboud University, P.O. Box 9010, 6500GL Nijmegen, The Netherlands
[5]LESIA - Observatoire de Paris, Université PSL, CNRS, Sorbonne Université, Université de Paris, 5 place Jules Janssen, 92195 Meudon, France

The data set we used consists in a time series of geomagnetic reversals $\zeta(t)$ which can be treated as a point process, namely $\zeta(t) = \delta(t-t_n)$ is different from zero only at discrete times t_n, when a reversal occurs (δ is the Kronecker function and $n = 1,\ldots,N$, being N the number of reversals in the dataset). The time sequence was collected and provided in Melott et al.[1] (2017) and allows us to investigate polarity reversals across the entire Phanerozoic eon. They utilised the latest geological timescale (2012) and the results of Hansma et al.[2] (2015) for the Devonian, as for this period no reliable data were available from the 2012 timescale. Polarity is considered as reverse when data values are 0, while it is normal when data values are 1. There are periods of rapid change, e.g., the Jurassic interval, as well as very long periods of fixed polarity, called superchrons and lasting for a few tens of Myr. We focus our analysis on the last 375 Myr since data are of higher quality than data predating this period. This is due to the difficulty of detecting unaltered sequences of rock suitable for magnetostratigraphic analysis older than 375 Myr[1]. During this time interval, a prolonged period of stable polarity, i.e., a superchron, has been found approximately between 121 and 83 Ma, usually known as the Cretaceous Normal Superchron (CNS), although the exact behaviour of the geomagnetic field during this interval remains unclear, as paleomagnetic data from different origin seem to show conflicting results[3]. A previous period of long stable polarity was the so-called Permo-Carboniferous Reversed Superchron (PCRS) between 262 and 318 Ma, which shows a high similarity with the CNS, in contrasting difference with periods of higher reversal rates[4]. This could suggest similar heat flux conditions in the CMB, which may favour compatible stability conditions throughout these magnetozones. However, both superchrons are still poorly understood and represent a challenge for the geomagnetic field models and for the investigation of their origin in relation to internal and external mechanisms[5].
From polarity reversals the time series of reversal rates has been extracted by averaging over sliding windows of length Δt = 8 Myr

$$\gamma(t) = \frac{1}{\Delta t}\int_{t}^{t+\Delta t} \zeta(t')\,dt' \qquad (1)$$

therefore $\gamma(t)$ measures how many reversals are present every 8 Myr.
The time evolution of $\gamma(t)$ has been analysed through the Empirical Mode Decomposition[6] (EMD, see the Methods section in the manuscript), a technique successfully used in several physical contexts[7-15]. From our time series we extracted

11 IMFs $C_j(t)$, reported in Fig. 2 of the manuscript along with the residual $r_m(t)$. The time averages (denoted hereafter by angular brackets) of the frequencies $\omega_j(t)$ allow us to define the typical periods $T_j = 2\pi/\langle\omega_j\rangle$ associated to each mode, reported in table (T1). We also calculated the probability density functions (reported in Fig. S1) of the instantaneous periods associated to each mode. As can be seen from Fig. S1 the periods are partially superposed, except for the last IMF C_{10}. This indicates either that there are no discrete periods or that they are masked by the non-stationarity, therefore the mean periods reported in Table T1 are only indicative of the quasi-periodicity associated to reversal rates. The non-stationarity of the physical process (namely the stochastic CMB heat flux driving) which induces strong fluctuations of the instantaneous frequency of the reversals rate is captured by the EMD analysis. Of course the paucity of the dataset at disposal makes difficult to unambiguously discriminate between different periods, if any exist, and to analyse the complexity of the phenomenon of geomagnetic reversals with data. The usual Fourier analysis, being based solely on the relative spectral peaks, cannot capture the richness of the non-stationarity of the phenomenon. The literature on the subject, which reports Fourier analysis of reversal rate, claims for the detection of various periodicities up to about 40 Myr, evidenced by single Fourier peaks within a background[1]. Our analysis, which is more appropriate for non-stationary processes, is compatible with the most common reported periodicities as far as the periods of the EMD modes with $j \leq 4$ are considered. For the modes with $j \geq 5$ we found longer periodicities, which in the usual Fourier spectra are perhaps masked by broadband low-frequency peaks. Among these long period modes, it is worth mentioning that the characteristic instantaneous periods of the modes C_9 and C_{10}, of about 130 and 240 Myr, respectively, are reminiscent of periods > 100 Myr sometimes found in the literature[16]. At these timescales the changing heat flux across the core-mantle boundary is expected to be influenced, for example, by variations in the flux of lithosphere subducted into the mantle[17].

The statistical significance of each IMF with respect to a white noise can be verified through a simple test based on the analytical determination of the mean square amplitude spread function for different confidence levels[18]. In fig. S2 we report the result of the test, which is based on the relation between the mean square modulus of each mode $E_j = \left\langle \left|C_j\right|^2 \right\rangle$ and the time period T_j. Since all the IMFs but C_0 exceed the theoretical dashed line which refers to a white noise process, this means that all the IMFs, apart for C_0, are significant with respect to a white noise at the 99th percentile. In other words, IMFs capture real quasi-periodic processes of the frequency of reversals, with different time scales T_j.
The potential functions U_j (see Methods section in the main manuscript) of all the EMD modes are reported in Fig. S3. Only two kind of potential shapes are roughly present in the dataset, namely single-well potentials for the set of modes $H = \{0 \leq j \leq 4\}$ and double-well potentials for the set of modes $L = \{5 \leq j \leq 10\}$.
Finally, by exploiting the orthogonality of IMFs, through their partial sums we can reconstruct the reversal rates which are responsible for a single-well potential as

$$\gamma_H(t) = \sum_{j=1}^{4} C_j(t) \tag{8}$$

(where IMF C_0 was not considered according to the statistical significance test) and distinguish them from those that are responsible for a double-well potential

$$\gamma_L(t) = \sum_{j=5}^{10} C_j(t) \tag{9}$$

From the two time sequences, we can obtain the average single-well and double-well potentials U_H and U_L reported in Fig. S4. A comparison between the partial reconstruction γ_L obtained by summing the IMFs of the set of modes $L = \{5 \leq j \leq 10\}$ (yellow curve) and a realisation obtained from the stochastic Langevin model (see main manuscript, green curve) is shown in Fig. S5, while in Fig. S6 the histograms of the stationary solution of the Fokker-Planck equation (see Methods in the main manuscript), the Langevin model, and the partial reconstruction γ_L are compared.

Table T1. We report the typical periods T_j, measured in Myr, associated to each EMD mode, obtained from the averages of the instantaneous frequencies (see text). Since the frequency ω_j of each mode is not constant, the errors on T_j have been calculated as the standard deviation of the average. The set of modes H = $\{0 \leq j \leq 4\}$ define chrons periods characterized by single-well potentials, while the set of modes $L = \{5 \leq j \leq 10\}$ define longer periods characterized by double-well potentials.

j-th EMD mode	Typical period T_j (Myr) associated to each EMD mode
0	3.7 ± 0.2
1	7.1 ± 0.5
2	13.7 ± 0.7
3	23 ± 1
4	29 ± 1
5	38 ± 2
6	46 ± 2
7	75 ± 3
8	95 ± 5
9	131 ± 6
10	240 ± 10

Figures

Fig. S1: Probability density functions of the instantaneous periods of the IMFs obtained through the EMD technique applied to the reversal rate time series.

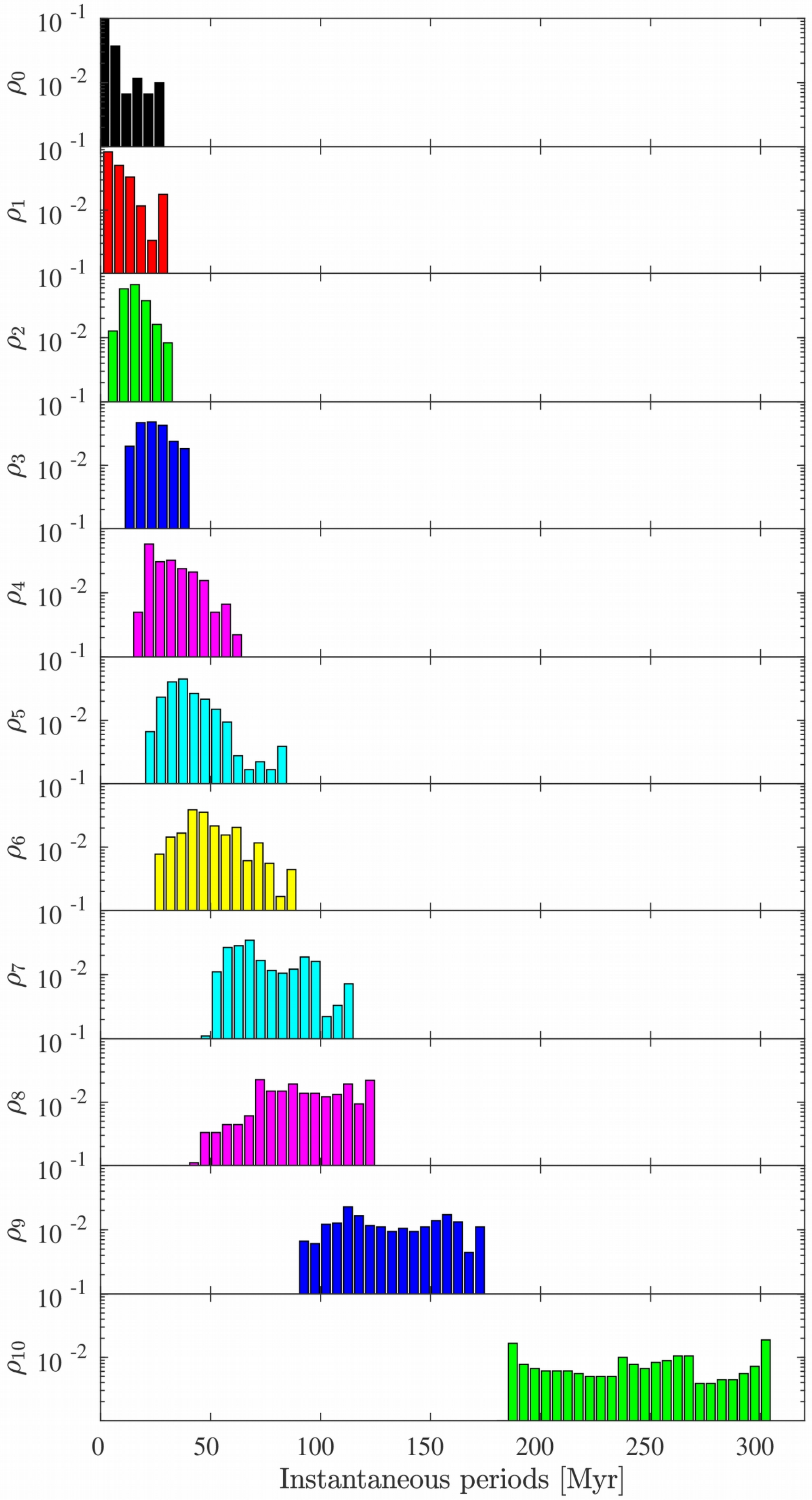

Fig. S2: EMD significance test obtained by plotting the normalised mean square modulus E_j/E_0 of each EMD mode (black bullets) as a function of the corresponding period T_j of the j-th mode. The dashed line corresponds to the 99th percentile for a white noise. All EMD modes, but the first one, are significant with respect to a white noise.

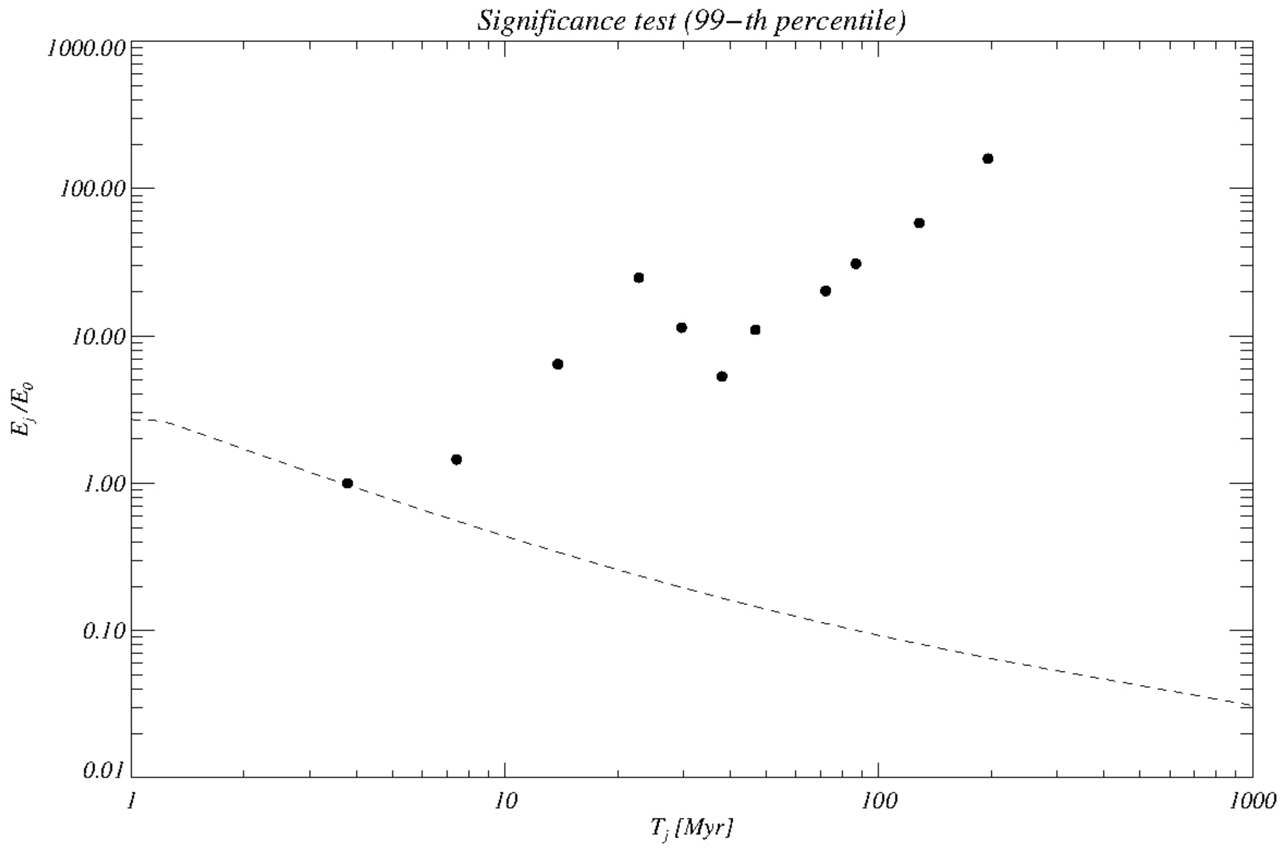

Fig. S3: Potentials U_j of all the EMD modes obtained by inverting the Fokker-Planck relation (see Methods section in the main manuscript), reported as a function of the standardized IMFs

$$C_j^{\sigma} = \{C_j - \langle C_j \rangle\} / \langle C_j^2 \rangle^{1/2}$$

The set of modes $H = \{0 \leq j \leq 4\}$ correspond to short chrons' periods and are characterized by single-well potentials, while the set of modes $L = \{5 \leq j \leq 10\}$ show longer periods and are characterized by double-well potentials.

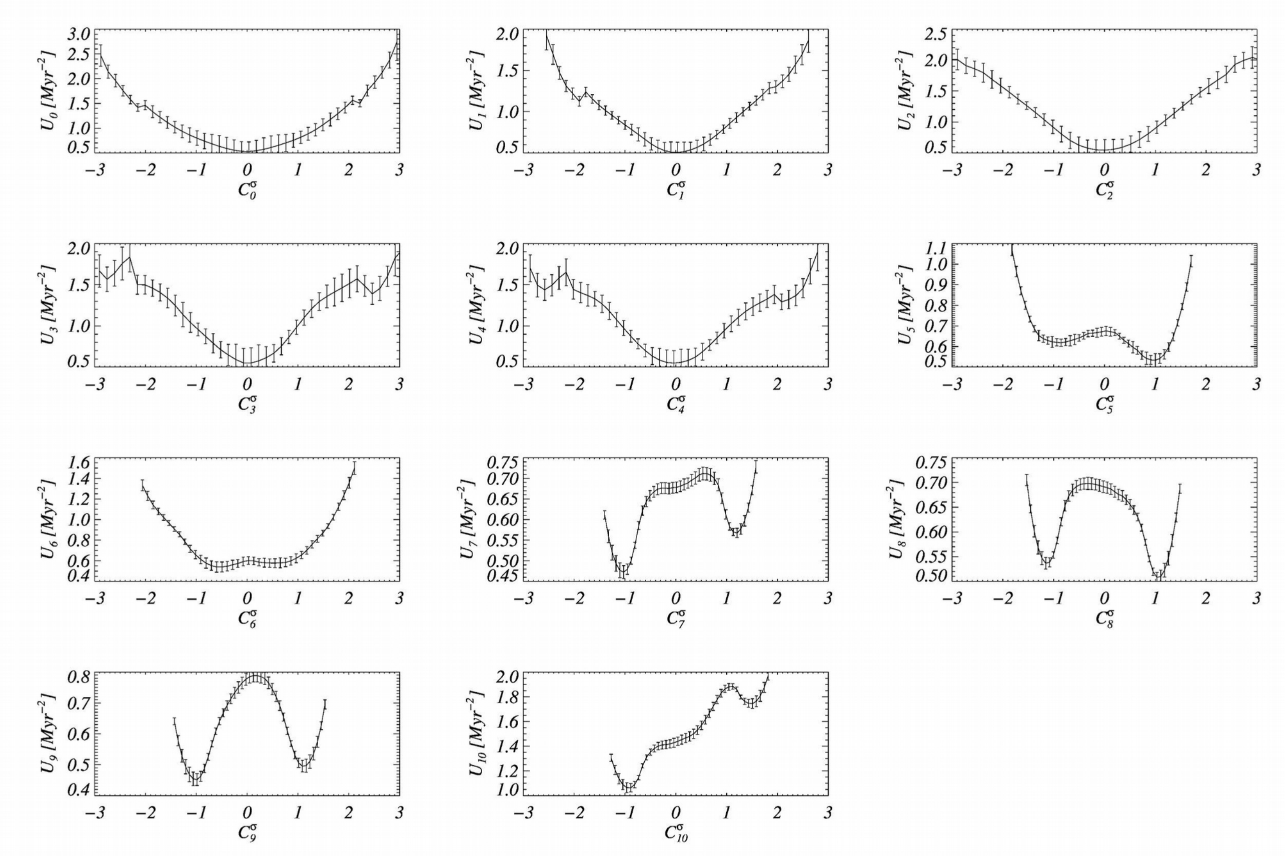

Fig. S4: Reconstructed reversal rates obtained by partial sums of the decomposition and resulting potentials. In the left panels we report the partial sum of reversal rates for the set of modes with $1 \leq j \leq 4$ (upper left panel), and the corresponding single-well potential (lower left panel) which represent average chron durations. In the right panels we report the partial sum of reversal rates for the set of modes $L = \{5 \leq j \leq 10\}$ (upper right panel) and the corresponding double-well potential (lower right panel) which reflects longer then average (superchron-like) periods. The red lines in the bottom panels are the best fit for both potentials obtained by using a second-order (left) and fourth-order (right) polynomial fit, respectively.

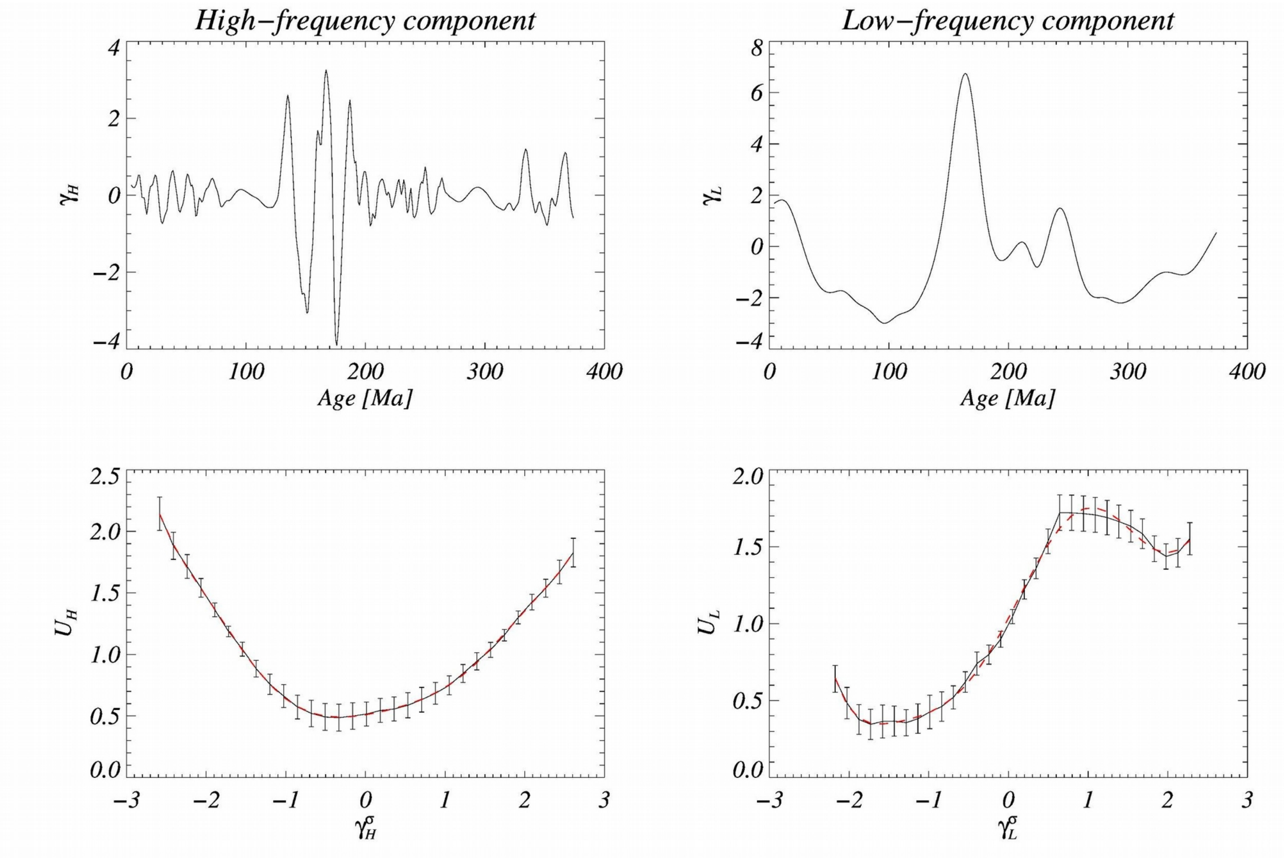

Fig S5: Comparison between the partial reconstruction γ_L obtained by summing the IMFs of the set of modes $L = \{5 \le j \le 10\}$ (yellow curve) and a realisation obtained from the stochastic Langevin model (green curve).

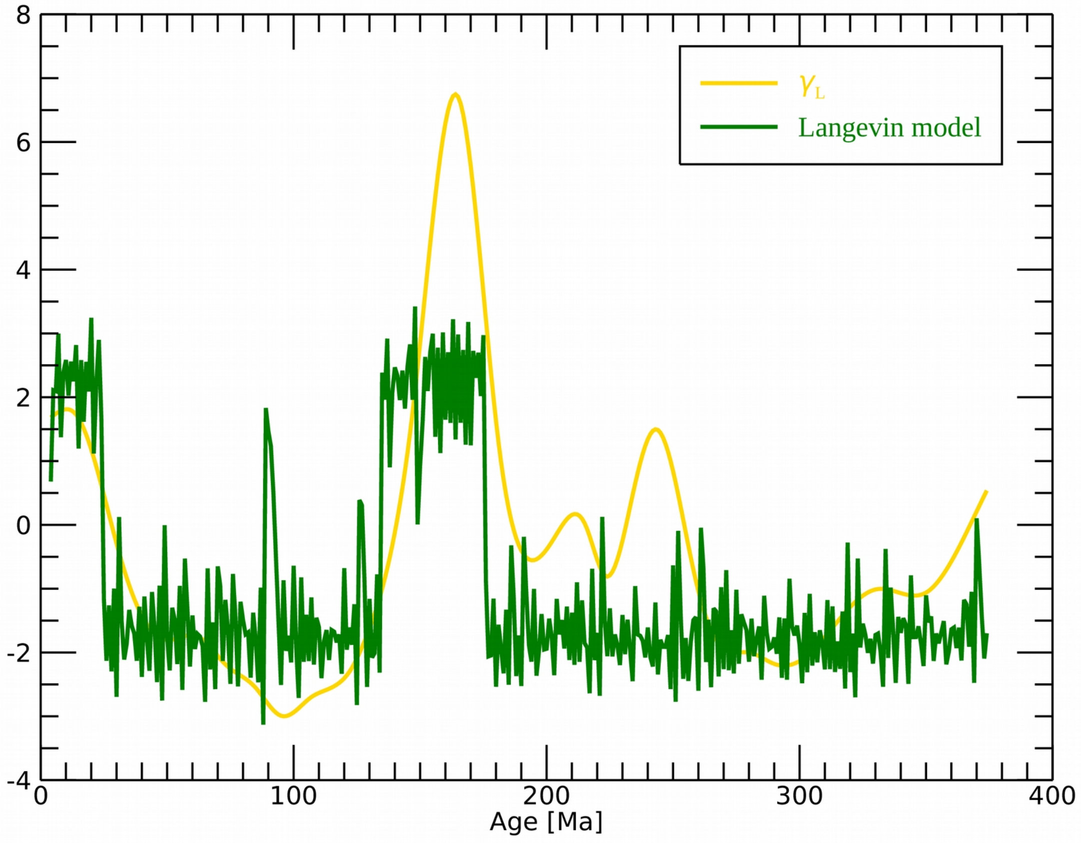

Fig. S6: Comparison between the histograms of the stationary solution of the Fokker-Planck equation (blue line), the Langevin model (green bars), and the partial reconstruction γ_L obtained by summing the IMFs of the set of modes $L = \{5 \leq j \leq 10\}$ (yellow bars).

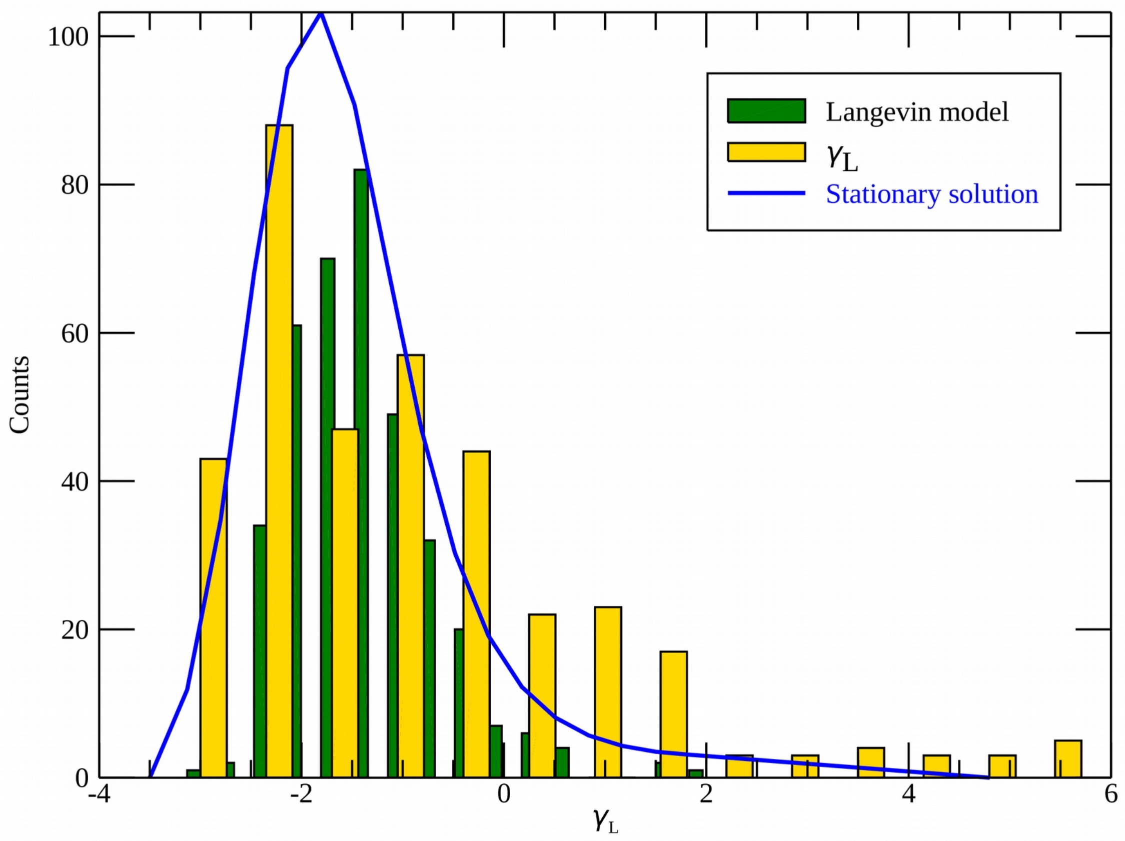

References

1. Melott, A.L., Pivarunas, A., Meert, J.G. & Lieberman, B.S. Does the planetary dynamo go cycling on? Re-examining the evidence for cycles in magnetic reversal rate. *International Journal of Astrobiology*, doi:10.1017/S1473550417000040 (2017).
2. Hansma, J. et al. Late Devonian carbonate magnetostratigraphy from the Oscar and horse spring ranges. Leonard Shelf, Canning Basin, Western Australia. *Earth Planet. Sci. Lett.* **40**, 232-242, (2015).
3. Granot, R., Dyment, J. & Gallet, Y. Geomagnetic field variability during the Cretaceous Normal Superchron. *Nature Geosci* **5,** 220–223 (2012) doi:10.1038/ngeo1404.
4. de Oliveira, W. P., Franco, D. R., Brandt, D., Ernesto, M., da Ponte Neto, C. F., Zhao, X., et al. Behavior of the paleosecular variation during the Permian-Carboniferous Reversed Superchron and comparisons to the low reversal frequency intervals since Precambrian times. *Geochemistry, Geophysics, Geosystems* **19**, 1035– 1048, (2018).
5. Jacobs, J. A. The cause of superchrons, *Astronomy & Geophysics* **42,** 6.30–6.31, (2001).
6. Huang, N. E., et al. The empirical mode decomposition and the Hilbert spectrum for nonlinear and non-stationary time series analysis, *Proc. Roy. Soc. Lond. A*, **454**, 903–995, (1998).
7. Cummings, D.A.T., et al. Travelling waves in the occurrence of dengue haemorrhagic fever in Thailand, *Nature* **427**, 344-347 (2004).
8. Jamsek, J., Stefanovska, A., McClintock, P.V.E. & Khovanov I.A. Time-phase bispectral analysis , *Phys. Rev. E* **68**, 016201 (2003).
9. Hutt, A., Daffertshofer A., & Steinmetz, U. Detection of mutual phase syncronization in multivariate signals and application to phase ensembles in chaotic data, *Phys. Rev. E* **68**, 036219 (2003).
10. Wu, Z., et al. The modulated annual cycle: an alternative reference frame for climate anomalies, *Clim. Dyn.* **31**, 823 (2008).
11. Vecchio, A., Laurenza, M., Carbone, V. & Storini, M. Quasi-biennial modulation of solar neutrino flux and solar and galactic cosmic rays by solar cyclic activity. *Astrophys. J. Lett.* **709** L1 (2010).
12. Capparelli, V., Vecchio, A. & Carbone, V. Long-range persistence of temperature records induced by long-term climatic phenomena. *Phys. Rev. E* **84** 046103 (2011).
13. Capparelli, V., Franzke, C., Vecchio, A., Freeman, M. P., Watkins, N. W. & Carbone, V. A spatiotemporal analysis of U.S. station temperature trends over the last century. *J. Geophys. Res. Atmos.* **118** 7427 (2013).
14. Alberti, T., et al. Natural periodicities and Northern Hemisphere–Southern Hemisphere connection of fast temperature changes during the last glacial period: EPICA and NGRIP revisited. *Clim. Past* **10**, 1751-1762 (2014).
15. Piersanti, M., Alberti, T., Bemporad, A. et al. Comprehensive Analysis of the Geoeffective Solar Event of 21 June 2015: Effects on the Magnetosphere, Plasmasphere, and Ionosphere Systems. *Sol. Phys.* **292** 169 (2017).
16. Driscoll, P.E. & Evans, D.A.D. Frequency of Proterozoic geomagnetic superchrons. *Earth Planet. Sci. Lett.* **437**, 9-14 (2016).
17. Hounslow, M. W., Domeier, M., Biggin, A. J. Subduction flux modulates the geomagnetic polarity reversal rate, *Tectonophysics*, **742**, 34 (2018)
18. Wu, Z. & Huang, N. E. A study of the characteristics of white noise using the empirical mode decomposition method. *P. Roy. Soc. Lond. A* **460** 1597 (2004).

19. Livina, V. N., Kwasniok, F. & Lenton, T. M. Potential analysis reveals changing number of climate states during the last 60 kyr. *Clim. Past* **6** 77 (2010).
